# Plant responses to decadal scale increments in atmospheric CO_2_ concentration: comparing two stomatal conductance sampling methods

**DOI:** 10.1007/s00425-020-03343-z

**Published:** 2020-01-16

**Authors:** Sven Peter Batke, Charilaos Yiotis, Caroline Elliott-Kingston, Aidan Holohan, Jennifer McElwain

**Affiliations:** 1 Biology Department, Edge Hill University, St. Helen’s Road, Ormskirk, L39 4QP UK; 2 Botany Department, Trinity College Dublin, College Green, Dublin 2, Dublin, Ireland; 3 School of Agriculture and Food Science, University College Dublin, Stillorgan Road, Belfield, Dublin 4, Dublin, Ireland; 4 School Biology and Environmental Science, University College Dublin, Stillorgan Road, Belfield, Dublin 4, Dublin, Ireland

**Keywords:** Climate change, Water loss, Growth chambers, IRGA, Porometer

## Abstract

**Main conclusion:**

Our study demonstrated that the species respond non-linearly to increases in CO_2_ concentration when exposed to decadal changes in CO_2_, representing the year 1987, 2025, 2051, and 2070, respectively.

**Abstract:**

There are several lines of evidence suggesting that the vast majority of C3 plants respond to elevated atmospheric CO_2_ by decreasing their stomatal conductance (*g*_s_). However, in the majority of CO_2_ enrichment studies, the response to elevated CO_2_ are tested between plants grown under ambient (380–420 ppm) and high (538–680 ppm) CO_2_ concentrations and measured usually at single time points in a diurnal cycle. We investigated *g*_s_ responses to simulated decadal increments in CO_2_ predicted over the next 4 decades and tested how measurements of *g*_s_ may differ when two alternative sampling methods are employed (infrared gas analyzer [IRGA] vs. leaf porometer). We exposed *Populus tremula*, *Popolus tremuloides* and *Sambucus racemosa* to four different CO_2_ concentrations over 126 days in experimental growth chambers at 350, 420, 490 and 560 ppm CO_2_; representing the years 1987, 2025, 2051, and 2070, respectively (RCP4.5 scenario). Our study demonstrated that the species respond non-linearly to increases in CO_2_ concentration when exposed to decadal changes in CO_2_. Under natural conditions, maximum operational *g*_s_ is often reached in the late morning to early afternoon, with a mid-day depression around noon. However, we showed that the daily maximum *g*_s_ can, in some species, shift later into the day when plants are exposed to only small increases (70 ppm) in CO_2_. A non-linear decreases in *g*_s_ and a shifting diurnal stomatal behavior under elevated CO_2_, could affect the long-term daily water and carbon budget of many plants in the future, and therefore alter soil–plant–atmospheric processes.

**Electronic supplementary material:**

The online version of this article (10.1007/s00425-020-03343-z) contains supplementary material, which is available to authorized users.

## Introduction

The global land vegetation is a key driver in the hydrological and energy processes on our planet. The transfer of water from the soil, through the plants and into the atmosphere is regulated by stomatal pores on the leaf surface (Brodribb and McAdam 2017) and accounts for up to 80–90% of terrestrial evapotranspiration in some biomes (Jasechko et al. 2013). There are several lines of evidence suggesting that the vast majority of C3 plants respond to elevated CO_2_ by decreasing their stomatal conductance (*g*_s_) and rates of transpiration and by increasing their assimilation rates (*A*) and overall water use efficiency (WUE) (Ainsworth and Rogers 2007). Under extreme heat and aridity, *g*_s_ can increase in response to elevated CO_2_ but a greater magnitude of *A* increase still results in improved WUE (Purcell et al. 2018). Anatomically, elevated CO_2_ has been shown to reduce stomatal density (Woodward 1987; Woodward and Kelly 1995; McElwain and Steinthorsdottir 2017) and in some cases alter stomatal pore size (decrease or increase), thereby reducing maximum *g*_s_ to water vapor (Franks and Beerling 2009; Xu et al. 2016; Lammertsma et al. 2011). Stomatal physiological and anatomical responses to elevated CO_2_ have been shown to be coordinated (Haworth et al. 2013) and are dependent on the growth environment of the plant (Curtis and Wang 1998) and the plant’s underlying degree of plasticity and/or capacity to physiologically acclimate (Stitt and Krapp 1999; Ainsworth and Long 2005). The synergistic/antagonistic effects of other abiotic (e.g., light, vapor pressure deficit [VPD], soil moisture, nutrients etc.) and biotic (e.g., competition, predation etc.) factors can thus substantially alter any predicted direct plant responses to elevated CO_2_ (Medlyn et al. 2001; Saxe et al. 1998).

Experiments that try to address the effect of elevated CO_2_ on soil–plant–atmosphere water-relations are therefore notoriously difficult to conduct and each study system [e.g., free-air-carbon-enrichment (FACE), greenhouse, laboratory and plant growth chamber] has advantages and disadvantages (Ainsworth et al. 2008; Ainsworth and Long 2005; Porter et al. 2015; Poorter et al. 2016). For example, CO_2_ concentrations in FACE systems have been shown to fluctuate substantially (Pepin and Körner 2002) and are usually shut down at night when the air is too still to ensure constant elevated CO_2_ treatment, whereas growth chamber environments allow much tighter CO_2_ control (Poorter et al. 2016) but poorly represent real field conditions. In most FACE studies, elevated CO_2_ conditions are controlled between 538–680 ppm (Purcell et al. 2018), whereas it is only in growth chamber studies where much tighter CO_2_ control can be achieved. It has been theoretically demonstrated by Konrad et al. (2008) that small incremental changes in atmospheric CO_2_ could result in a non-linear *g*_s_ response to CO_2_, as opposed to a linear response that is currently assumed in Earth System Models. It is therefore important to not just compare plant responses to large, century changes in CO_2_ in in situ and ex situ experiments, as currently done in many FACE (Purcell et al. 2018) and chamber experiments, but also to consider investigating *g*_s_ responses to smaller, decadal changes in CO_2_ concentration predicted for the next 5–30 years (IPCC 2014).

Measuring the effect of elevated CO_2_ on plant physiological traits such as *g*_s_ is of global significance in understanding current, past and future plant responses to a changing climate (Betts et al. 2007; Huntington 2008; McElwain and Steinthorsdottir 2017; Chung et al. 2007; Gornish and Tylianakis 2013). However, collecting data on *g*_s_ across a wide variety of taxonomic groups, biomes and treatments is often hampered by costs and time constraints. Commercially available systems such as hand-held porometers and portable infrared gas analyzers (IRGA) are amongst the most commonly used devices to measure *g*_s_. However, the different approaches to gas exchange measurement make them suitable for different purposes. Leaf porometers measure *g*_s_ by placing the conductance of a leaf in series with two known conductance elements, and comparing the humidity measurements between them to estimate water vapor flux. Usually a leaf measurement takes 30 s during which an algorithm can predict the final *g*_s_ reading that would be achieved if unlimited time were allowed for true steady state conditions to occur (Decagon-Devices 2005). In the case of gas analyzers, a reference air mixture is continuously passed through the leaf chamber. Measurements of *g*_s_ are based on differences in H_2_O in the air streams that flow into and out of the leaf cuvette. In other words, the rate of water loss is used to calculate the rate of *g*_s_ (PP-Systems 2007). Since both systems are frequently used in physiological sample protocols in natural, semi-controlled and controlled environments (Long et al. 1996; Lüttge et al. 1986; Yiotis et al. 2017; Bakker 1991; Murray et al. 2019), it is imperative that any deviations in the results between these methods are considered; particularly when interpreted in the light of global change biology (Midgley et al. 1997). Surprisingly, little published data are available comparing porometer and gas analyzer systems (Murray et al. 2019) and, to our knowledge, no published comparison exists that has investigated the difference in derived results under controlled growth chamber environments.

Another fundamental issue that plant biologists face when taking measurements of *g*_s_ is that *g*_s_ fluctuates diurnally. Under natural conditions, maximum operational *g*_s_ is often reached in the late morning to early afternoon, with a mid-day depression around noon (Roessler and Monson 1985; Pathre et al. 1998). Konrad et al. (2008) theoretically demonstrated that maximum daily *g*_s_ can shift by approximately 1 h for every 180 ppm increase in CO_2_. The practical implications of this can be quite significant, particularly in studies that aim to identify maximum operational *g*_s_ responses to different experimental treatments such as elevated CO_2_. Although diurnal measurements have the disadvantage of being more time consuming and are restricted in the sense that they require the use of a gas analyzer, they do provide a better account of whole-physiological diurnal plant responses than porometers.

The aims of this study were to investigate *g*_s_ responses to simulated decadal increments in CO_2_ predicted over the next 4 decades (IPCC 2014) and to test how measurements of *g*_s_ may differ when two alternative sampling methods are employed (infrared gas analyzer [IRGA] vs. leaf porometer). We focused on both the differences between the recorded values using the two methods and the time-shifts of the maximum daily *g*_s_ value under fluctuating CO_2_. In addition, to compare and build the relationship between gas analyzer-collected and porometer-collected data, a data set of *g*_s_ from 47 species measured under natural field conditions was used from Murray et al. (2019) and compared to our chamber measured plants. In all cases stomatal conductance was measured with both sampling devices.

## Materials and methods

### Controlled-environment experiment

A total of 54 individuals of bare-rooted *Sambucus racemosa* L. (red elderberry), 54 saplings of *Populus tremula* L. (common aspen) and 18 saplings of *Populus tremuloides* Michx. (quaking aspen) were purchased and grown in controlled experiments. It was not possible to source more individuals of *P. tremuloides* due to very strict importation regulations. These species were chosen as they are each known to occur in more than one global biomes (boreal forest, temperature deciduous forest, temperate grassland/chaparral and temperate rainforest) (Murray et al. 2019) and have also been used in previous CO_2_ enrichment studies (Bernacchi et al. 2003). All plants were re-potted into 5 L pots using a growing medium comprising 90% Shamrock**®** Multi-Purpose Compost (Scotts Horticulture Ltd., Co. Kildare, Ireland) and a 10% combination of Perlite Standard 2–5 mm (Sinclair Pro, Cheshire, UK) and 3 g/l Osmocote® Exact Standard 12**–**14 M slow release fertilizer (15-9-11 + 2MgO + TE; Scotts International BV, Netherlands). The plants were kept for 2 weeks in the open air at Rosemount Environmental Research Station, University College Dublin (UCD), Ireland, before being treated for pests with an emulsifiable concentrate containing 5% pyrethrin (Pyrethrum 5EC at 20 ml/5 L—Agropharm Ltd., UK). The plants were then moved into CONVIRON (Winnipeg, Manitoba, Canada) BDR-16 and BDW-40 plant growth chambers within the Programme for Experimental Atmospheres and Climate (PÉAC) facility at UCD. The chambers allow monitoring and control of atmospheric conditions including air temperature (*T*) (°C), relative humidity (RH) (%), light (PAR) (µmol m^−2^ s^−1^) and atmospheric [O_2_] (%) and [CO_2_] (ppm). For the experiment, chambers were programmed to run a 16.5 h/7.5 h day/night cycle. Maximum day time *T* and RH were set to 22 °C and 70%, respectively. Maximum night time *T* and RH were set to 15 °C and 60%, respectively. Light intensity was set to reach a maximum of 600 μmol m^−2^ s^−1^ at noon (Table 1) and O_2_ concentration was set to ambient concentration of 20.9% in all chambers. A ramping program was used to ensure a uniform diurnal increase in *T*, RH and light conditions. CO_2_ concentrations were set to 350, 420, 490 and 560 ppm in two chambers per CO_2_ treatment (8 chambers in total) and were monitored in each chamber with a PP-Systems WMA-4 CO_2_ gas analyzer. The CO_2_ concentrations represented the year 1987, 2025, 2051 and 2070, respectively, according to the low-medium stabilization RCP4.5 scenario (IPCC 2014). Supplementary CO_2_ for all the chambers was provided by a compressed gas tank containing liquid CO_2_. Since two different chamber types were used in the experiment, an additional control chamber (BDW-40) was added to the 420 ppm treatment to identify any potential confounding effects that might have occurred due to differences in chamber type (Porter et al. 2015). To attain sub-ambient (350 ppm) CO_2_ in the chambers, an inline fan with a variable damper regulated the amount of air that was passed from the chambers through an external soda lime unit (2–5 mm Sofnolime™—Molecular Products Group Ltd., Essex, UK). The CO_2_ free air was then passed back into the chambers and CO_2_ was injected to reach the target sub-ambient set point conditions. All measured chamber conditions are reported in Table 1.

**Table 1:** Plant growth chamber parameter settings

	Type		CO_2_ set point	CO_2_ measured	Light	Temp. day	RH day	Temp. night	RH night
		Set point	350–560 ppm		600 µmol	22 °C	70%	15 °C	60%
Chamber 1–2	BDW 40	Mean	350	345.59	600.08	20.46	68.12	16.28	65.86
	(*n* = 2)	SD		34.27	30.72	0.27	3.36	0.27	3.42
Chamber 3–5	BDW 40/BDR 16	Mean	420	434.06	551.60	20.41	67.44	16.24	66.01
	(*n* = 1/*n* = 2)	SD		19.09	16.83	0.54	5.56	0.73	6.22
Chamber 6–7	BDR 16	Mean	490	481.76	568.87	20.58	68.75	16.66	64.14
	(*n* = 2)	SD		23.26	19.70	1.04	6.86	2.43	9.82
Chamber 8–9	BDR 16	Mean	560	544.34	560.09	20.45	69.95	16.27	66.10
	(*n* = 2)	SD		30.96	25.74	0.38	6.74	0.53	7.74

To acclimatize plants to chamber conditions, six plants of *P. tremula* and *S. racemosa* and two plants of *P. tremuloides* were transferred into each chamber (Table 2) and grown under ambient local conditions (420 ppm CO_2_ and 21% O_2_) for 14 days before treatment conditions were initiated. The plants were then grown for 126 days under treatment. Plants were watered and fertilized (N:P:K; 22:4:22) every 2 days and 2 weeks, respectively. Soil moisture content was monitored using a Delta-T Devices HH2 Moisture Meter (Delta-T Devices Ltd., Cambridge, UK) (*P. tremula* = 0.25 ± 0.12 m^3^·m^−3^; *P. tremuloides* = 0.29 ± 0.11 m^3^·m^−3^; *S. racemosa* = 0.25 ± 0.13 m^3^·m^−3^). Plants were rotated randomly twice within each chamber to avoid spatial acclimation (Hammer and Hopper 1997) or within-chamber variability (Porter et al. 2015).

**Table 2:** Number of individuals grown under different CO_2_ conditions and chamber types

	CO_2_ (ppm)	Type	*S. racemosa*	*P. tremula*	*P. tremuloides*
Chamber 1–2	350	BDW 40	12	12	4
Chamber 3	420	BDW 40	6	6	4
Chamber 4–5	420	BDR 16	12	12	2
Chamber 6–7	490	BDR 16	12	12	4
Chamber 8–9	560	BDR 16	12	12	4
Total			54	54	18

Stomatal conductance was measured using a PP-Systems Ciras-2 infrared gas analyzer attached to a PLC6(U) Automatic Universal Leaf Cuvette and a hand-held Decagon Devices SC-1 Leaf Porometer. Measurements were taken inside the chambers on a minimum of two, fully expanded leaves per individual plant that had developed fully under treatment conditions. Each leaf was labelled and measured repeatedly throughout the experiment. The IRGA was used to measure *g*_s_ over 24 h for each species in all treatments (*n* = 10), except the 490 ppm treatment as we were restricted by the availability of equipment. The light emitting diode (LED) unit was removed from the leaf cuvette to attain ambient light conditions in the cuvette (see supplementary figure). Leaves were allowed to equilibrate within the cuvette for a minimum of 20 min. until *g*_s_ had remained stable for approximately 15 min. at a VPD of 1 kPa. CO_2_ concentrations in the leaf cuvette were fixed to the ambient treatment condition of each of the growth chambers (Table 1). Leaf temperature was determined using the energy balance setting. To avoid temporal differences between measurements, plants were measured in rotation across treatments. In combination with the 24 h measurements, a leaf porometer was used to make additional spot measurements at 11 am in the morning on two leaves per individual plant over several days. To ensure plants were measured at the same time of the day, the time setting of the plant growth chambers was staggered by 1 hour.

### Field data

See Murray et al. (2019) for a detailed account of the field data collection protocol. Briefly, *g*_s_ measurements were carried out in North and Central America in the summer of 2014. A total of 47 C3 woody angiosperm tree and shrub species were sampled in two boreal forest sites (Bird Creek [60°58′ N, 149°28′ W] and Kenai [60°33.3′ N, 151°12.8′ W], Alaska, USA), one temperate deciduous forest site (Smithsonian Environmental Research Centre [38°53′ N, 76°32′ W], Maryland, USA), two tropical seasonal forest (wet) sites (Cambalache [18°27′ N, 66°35′ W] and Guajataca [18°24′ N, 66°58′ W], Puerto Rico) and one tropical seasonal forest (dry) site (Guanica [17°93′ N, 66°92′ W], Puerto Rico). Stomatal conductance was measured with a CIRAS-2 gas analyzer (PP-Systems, Amesbury, MA, USA) attached to a PLC6(U) cuvette fitted with a 1.7 cm^2^ measurement window and a red/white light LED unit. Stomatal conductance was measured at ambient atmospheric CO_2_ of 400 ppm on an average of four individuals per species between 9:00 am and 13:00 pm on a sun exposed leaf following standard sample protocols (Berveiller et al. 2007; Domingues et al. 2010; Koch et al. 2004; Rowland et al. 2015; Dang et al. 1997). Cuvette conditions were set at 200 cm^3^ min^−1^ air flow, 1000 μmol m^−2^ s^−1^ light intensity and 80–90% incoming mole fraction of water vapor. To standardize our measurement protocol for each site, regardless of the temperature changes during the daily measurement time window, they calculated the average site-specific leaf temperature at 9:00 am by recording the leaf temperature of at least ten leaves belonging to ten different species grown at each site. For the final *g*_s_ measurements each leaf was left to equilibrate for at least 15 min before values were recorded.

In addition to the IRGA *g*_s_ measurements, a Decagon Devices SC-1 steady state Leaf Porometer was used to measure *g*_s_ on the same species and site, on fully exposed leaves (Murray et al. 2019). One leaf on three individuals per species was measured consecutively over 4 days. As with the IRGA, measurements were not taken on wet days or on wet leaves. Where moisture was a factor, excess moisture was blotted off and the leaf was left to dry before it was measured (Murray et al. 2019).

### Analysis

Data were tested for normality and equal variance. Difference in *g*_s_ between treatments was tested separately for each species using ANOVA comparison. The ANOVA was weighted by the soil moisture content of each plant to account for variability in soil moisture between *g*_s_ measurements. ANOVA comparisons that were significant were further analyzed using pairwise tests with Bonferroni corrections. To identify how maximum diurnal *g*_s_ shifted between treatments, a polynomial surface was fitted using non-parametric locally weighted regression. Maximum *g*_s_ was then calculated for each fit (species and treatment) separately. To test for chamber effects as a result of using different chamber types, mixed effect models were used. All analysis was performed using the statistical package ‘R’ version 3.4 (R Developing Core Team 2017).

## Results

**Fig. 1:**
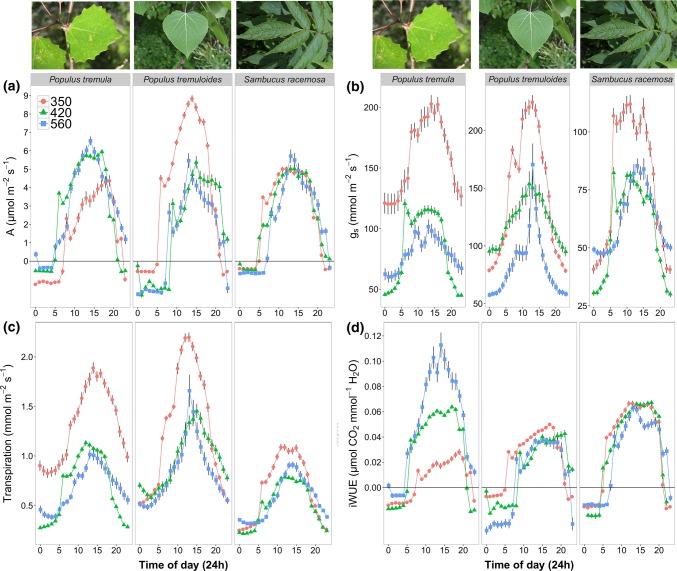
Diurnal physiological responses of plants grown under 350 (circle and red), 420 (triangle and green) and 560 ppm (square and blue) CO_2_. No IRGA data for the 490 ppm treatment was collected due to restricted access to equipment. **a** assimilation (*A*), **b** stomatal conductance (*g*_s_), **c** transpiration and **d** intrinsic water use efficiency (iWUE). Each value is the mean of approximately ten measurements per treatment (*n* = 10). Vertical bars represent the 95% confidence interval

In the chambers, diurnal physiological responses of photosynthetic assimilation (*A*) (µmol m^−2^ s^−1^), *g*_s_ (mmol m^−2^ s^−1^), transpiration (mmol m^−2^ s^−1^) and iWUE (= *A*/*g*_s_) (µmol m^−2^ s^−1^)/(mmol m^−2^ s^−1^) contrasted between and within species grown at different CO_2_ concentrations (Fig. 1). Assimilation was lowest at the 350 ppm treatment for *P. tremula* and higher at the 420 and 560 ppm treatments (Fig. 1a); iWUE was therefore greatest for individuals grown under 560 ppm conditions (Fig. 1d). Assimilation and iWUE for *P. tremuloides* showed the opposite response with higher *A* and iWUE under 350 ppm CO_2_ conditions. Although *A* and iWUE were also higher for *S. racemosa* in the 350 ppm CO_2_ treatment, the differences between treatments were much smaller (Fig. 1a, d).

**Fig. 2:**
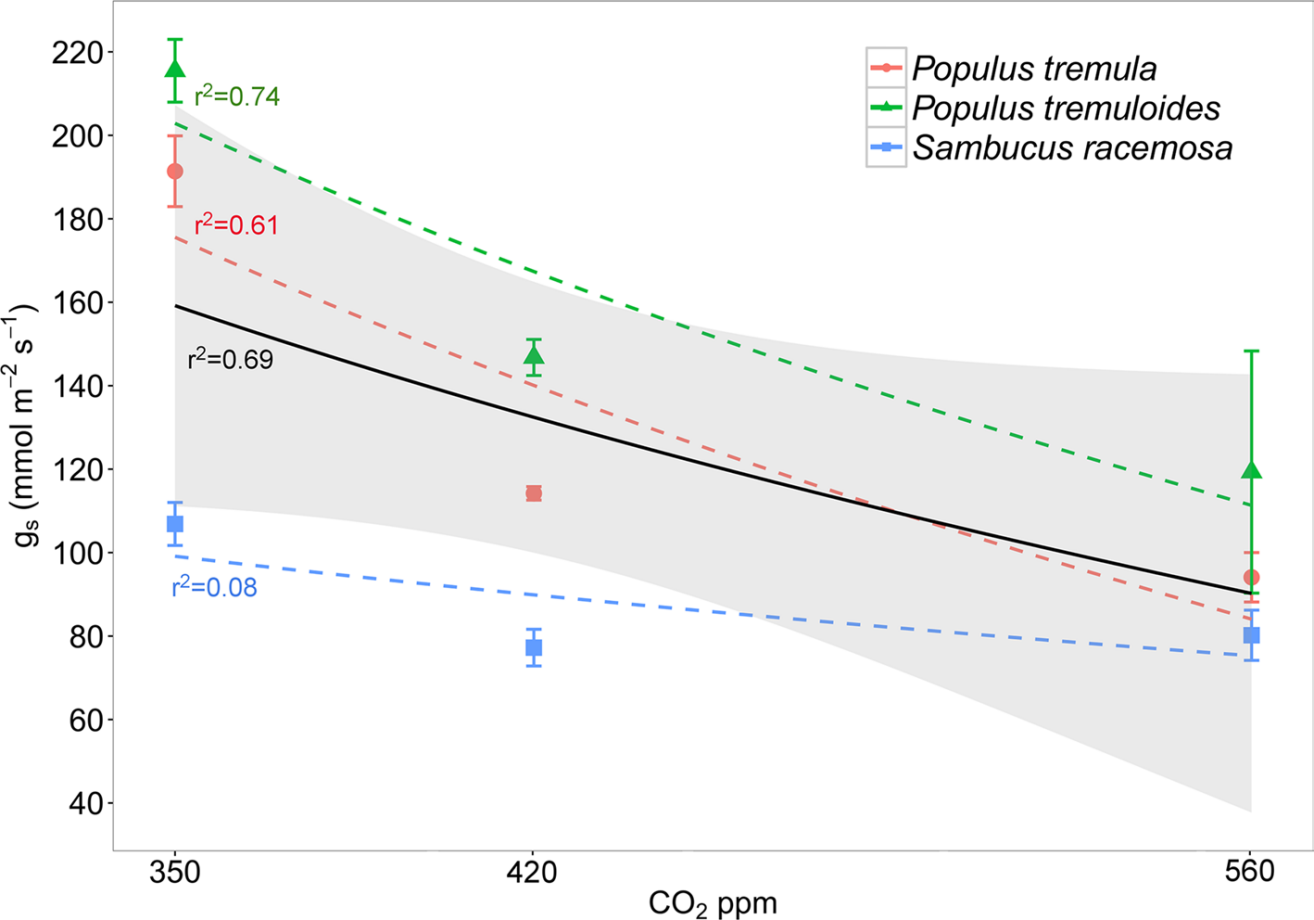
Fitted species and mean (black solid line) log regression (*F* = 6.825, *r*^2^ = 0.69, *p* = 0.03) of *g*_s_ across CO_2_ treatments. Grey-shaded area represents the standard error for the mean fit of all three species. Individual measurements represent the mean and 95% confidence intervals of daytime *g*_s_ measured using a PP Systems CIRAS-2 (*n* = 10)

**Fig. 3:**
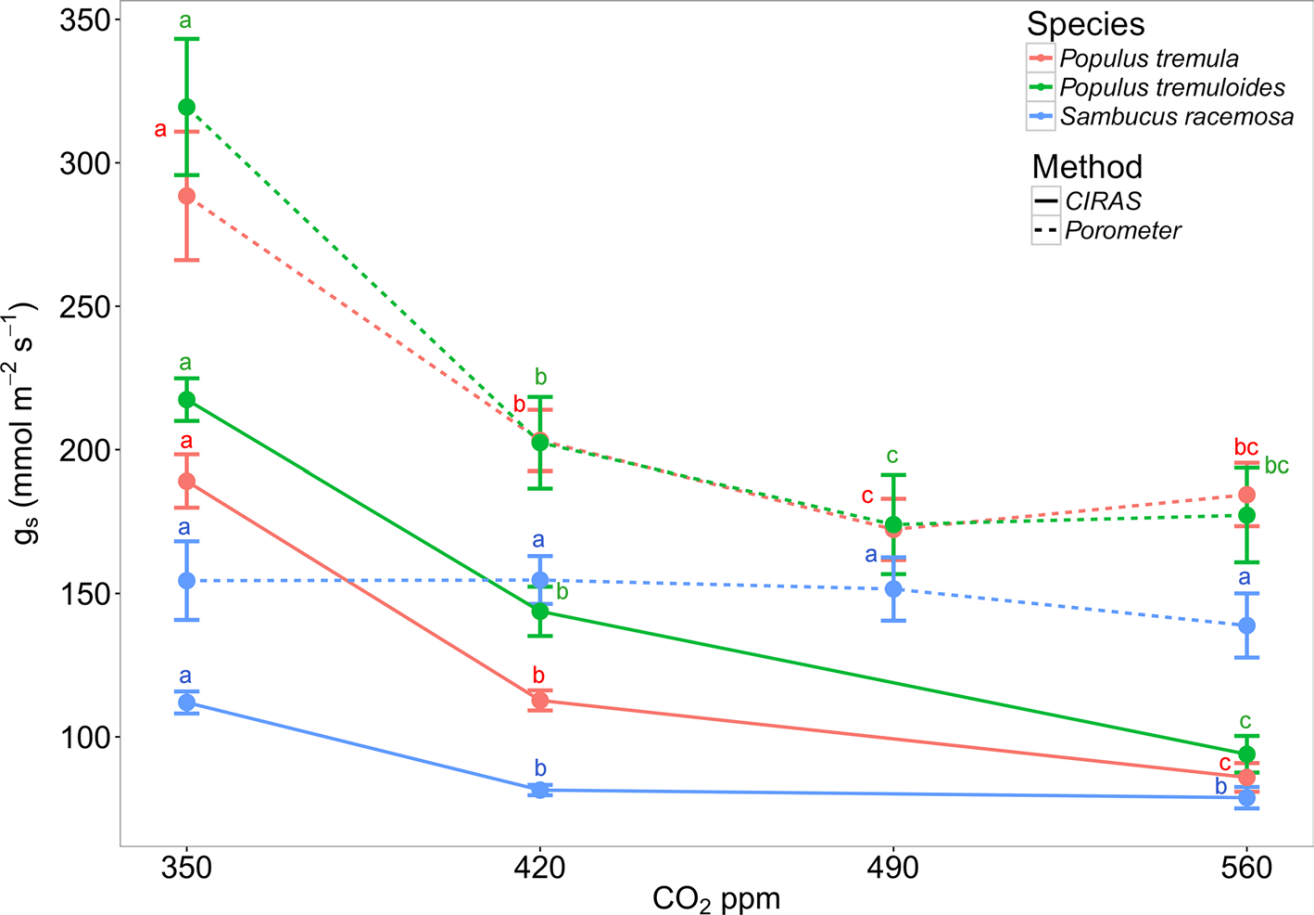
Stomatal conductance measured with a porometer (dashed lines) and an IRGA (solid lines) for each species (different colors) and within each CO_2_ (ppm) treatment. The means and 95% confidence intervals are shown. Different letters indicate significant differences between treatments (Bonferroni post-hoc test at *p* < 0.05). The spot measurements were taken between 11 am and noon every second day. Each value is the mean of approximately 160 (porometer) and 10 (IRGA) repeated measurements per treatment

Generally, *g*_s_ and transpiration decreased under elevated CO_2_ (Fig. 1b, c) for all three species, with the greatest difference observed between the 350 ppm and 560 ppm CO_2_ treatments. The decrease in *g*_s_ under elevated CO_2_ was best explained by a log regression (overall *F *= 6.825, *r*^2^ = 0.689, *p* = 0.03), showing that the mean *g*_s_ response to CO_2_ when measured at 11 am was non-linear for all three species in the IRGA measurements (Fig. 2). This non-linear decrease was also reflected for two species (*P. tremula* and *P. tremuloides*) in the porometer measurements (Fig. 3).

**Fig. 4:**
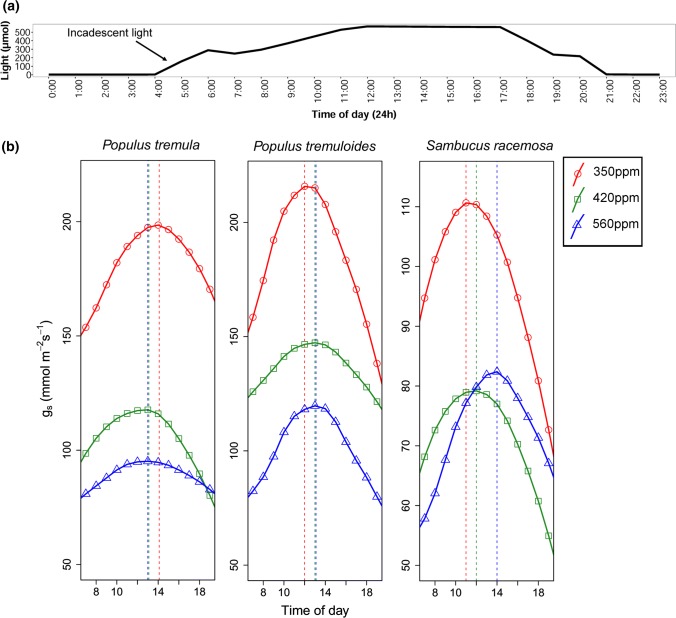
**a** Diurnal chamber light pattern. **b** Diurnal non-parametric locally weighted polynomial regression curves of *g*_s_ calculated for plants grown under 350 (circle and red), 420 (square and green) and 560 ppm (triangle and blue) CO_2_. The dashed lines represent the maximum *g*_s_ at time *t*

The time of the maximum operational *g*_s_ also differed between treatments and was species-specific (Fig. 4b). For *S. racemosa*, the maximum operational *g*_s_ shifted across the day by 1–2 h for each 70 ppm increase in CO_2_. For *P. tremuloides*, maximum operational *g*_s_ shifted across the day by 1–2 h from 350 to 420 ppm CO_2_ but no shift was observed between 420 and 560 ppm CO_2_. There was a shift in maximum *g*_s_ to earlier hours for *P. tremula* in response to changes in CO_2_ concentrations (Fig. 4b).

The comparison of measured *g*_s_ between the porometer and IRGA in the growth chambers showed that, in general, the measured *g*_s_ responses under elevated CO_2_ were proportionally very similar. Both methods detected a decrease in *g*_s_ with elevated CO_2_. However, the magnitude of measured *g*_s_ responses varied between the porometer and CIRAS, with an average of ~ 25% higher values in *g*_s_ observed when the porometer was used (Fig. 3). Although no statistically significant difference (*p* > 0.05) was observed between the three treatments, the relative difference in measured *g*_s_ between the porometer and the IRGA increased with elevated CO_2_ (~ 20–30%).

**Fig. 5:**
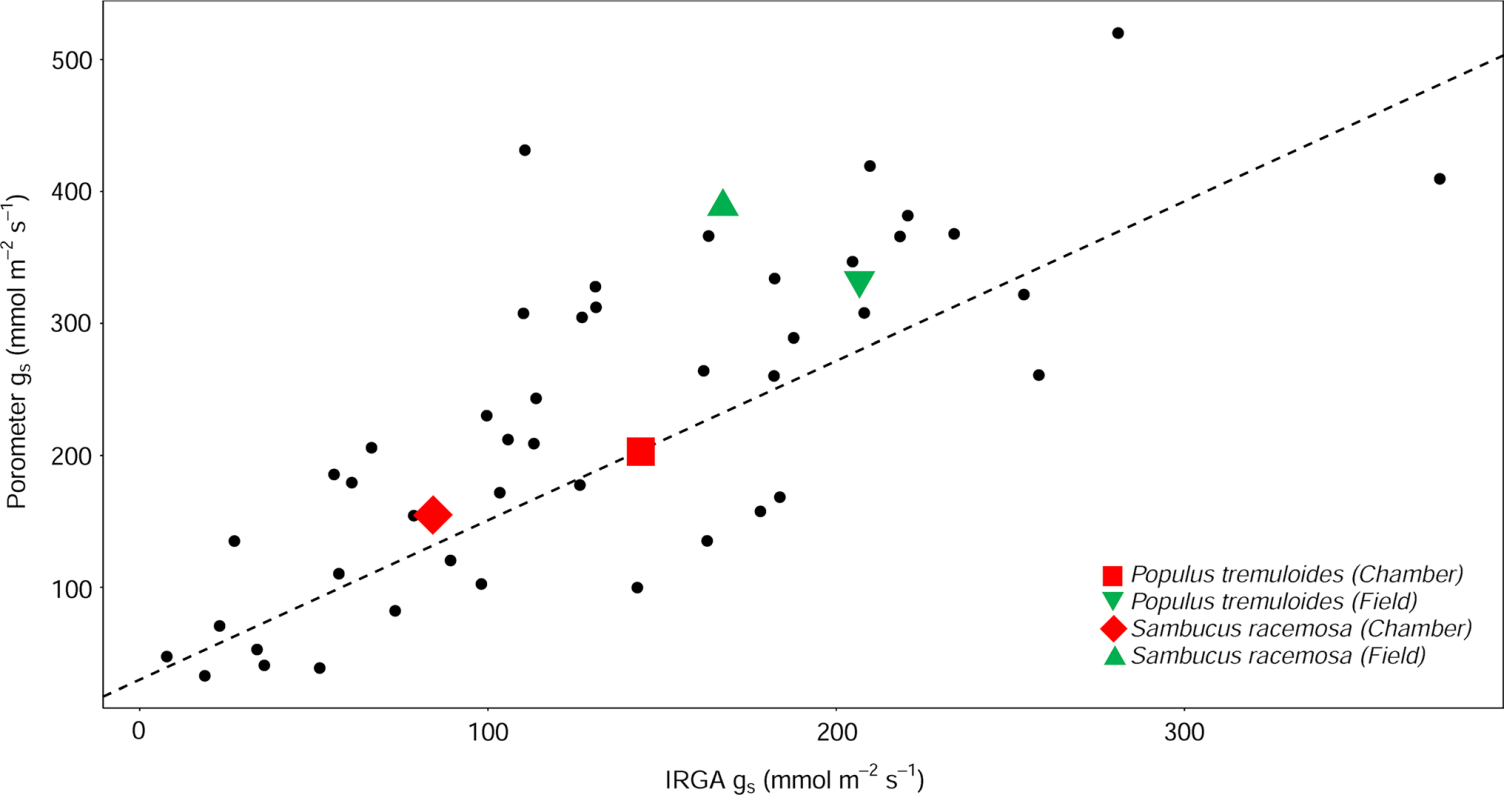
Comparison of mean porometer and IRGA measured *g*_s_ in field and chamber conditions. The black dots show the porometer and IRGA comparison of 45 species in the field by Murray et al. (2019). The green symbols show the same comparison in the field for *P. tremuloides* (upside down triangle) and *S. racemosa* (upright triangle). The red symbols show the porometer and IRGA comparison in the chambers for *P. tremuloides* (square) and *S. racemosa* (diamond). Note that the field measurements were done under 400 ppm, whereas the chamber measurements for this comparison was done with individuals in the 420 ppm treatment. The *y*-intercept was fixed to 30 mmol m^−2^ s^−1^. This was done because preliminary measurements using dry pieces of paper and plastic revealed that porometer measurements were on average 30 mmol m^−2^ s^−1^ higher from zero compared to IRGA measurements (*g*_s_ = 0) [see Murray et al. (2019) for more detail]

Following the work of Murray et al. (2019), there was a strong positive correlation between the IRGA and the porometer measured *g*_s_ of our 47 measured species in the field (Fig. 5; *F* = 64.05, *r*^2^ = 0.58, *p* < 0.01). Figure 5 also suggests that low conducting species display greater proportional difference between IRGA and porometer observations due to the fact that the relationship crosses the *y* axis at a value of 30 mmol m^−2^ s^−1^ (*F* = 7.29, *r*^2^ = 0.12, *p* < 0.01). The mean differences in measured *g*_s_ between the IRGA and the porometer for *P. tremuloides* and *S. racemosa* measured in the field in woody C3 angiosperm taxa was 24.3% and 40.6%, respectively. This was of a similar magnitude compared to the difference of *g*_s_ between the IRGA and the porometer for *P. tremuloides* (17%) and *S. racemosa* (31%) measured in the growth chambers at 420 ppm (Fig. 5).

## Discussion

The non-linearity of *g*_s_ response to increasing CO_2_ has been predicted in empirical explorations and modelling studies (Konrad et al. 2008; Maherali et al. 2002; Gill et al. 2002; Medlyn et al. 2011; de Boer et al. 2011). However, in many published studies plants are often exposed to large step increases in CO_2_, mostly comparing ambient to high (~ 600 ppm) CO_2_ manipulations; representing centurial increase in CO_2_ (Ainsworth et al. 2008; Ainsworth and Long 2005). Since atmospheric CO_2_ is expected to increase gradually in the future, large step increases from CO_2_ studies are not easily extrapolated to intermediate CO_2_ concentration increases; hence why in some studies a non-linear response has often gone undetected (Long et al. 2004). The non-linear decrease in *g*_s_ can only be detected when plants are exposed to decadal rather than centurial magnitude CO_2_ change. Growth chamber experiments compared to other experimental systems (e.g., FACE) have a technical advantage when it comes to subtle (e.g., decadal) manipulations of the CO_2_ environment, as they can control CO_2_ more tightly (increments of + 50 to + 70 ppm are possible, see Table 1). We found clear evidence that at least two of the species measured in this experiment are responding to elevated CO_2_ with a non-linear decrease in *g*_s_. Understanding and demonstrating this non-linear decrease through experimental manipulation can help to refine predictions to ecosystem responses (Bonan et al. 2014).

The decrease in *g*_s_ under elevated CO_2_ was in some cases accompanied by an increase in *A* and iWUE (Fig. 1). The adjustment of *g*_s_ to CO_2_, via feedback regulation of stomatal aperture and or stomatal density and pore size, is part of the mechanism for optimizing CO_2_ uptake with respect to water loss (Haworth et al. 2013). The decrease of *A* in *P. tremuloides* under elevated CO_2_ can possibly be attributed to a strong down-regulation of photosynthesis (Ainsworth and Long 2005) or increased stomatal limitation (e.g., speed or stomatal anatomy) compared to the two other species. The latter is less likely to be the case, as *g*_s_ in *P. tremuloides* at 420 and 560 ppm are equal or higher compared to the *g*_s_ values of the other two species. In *S. racemose,* where *A* did not change and *g*_s_ was generally lower compared to the other species, the possible increased stomatal limitation at elevated CO_2_ had potentially a greater impact on *A* values. When plotting *A* against *g*_s_ for each species, the species that had the strongest control/limitation of *g*_s_ on *A* was *P. tremuloides*, followed by *P. tremula* and *S. racemosa* (not shown). Furthermore, it has been demonstrated that long-term exposure to CO_2_ in C3 plants can, in some instances, result in a reduction or even complete suppressing of *A* (Makino and Mae 1999; Faria et al. 1996). Such responses have been attributed to secondary responses related to decreased nitrogen content or excess carbohydrate accumulation in the leaves, and has often been documented in drought conditions (Cregger et al. 2014; Griffin et al. 2004). Drought is less likely to be the cause of the responses observed here, as the soil moisture was monitored when *g*_s_ measurements were taken throughout the experiment and did not change between treatments (*p* > 0.05) or throughout the duration of the experiment. Also, sink limitations as a result of pot experiments are a likely cause of the observed down regulation of *A* in *S. racemosa* under elevated CO_2_ (Ruiz-Vera et al. 2017; Schaz et al. 2014). All plants were provided with liquid fertilizer and were potted in soil that contained slow release fertilizer. However, no soil characteristics (e.g., nitrogen, pH) were measured to exclude this possibility. *S. racemosa* grew much faster compared to the two other species (pers. obs.), which could have resulted in less optimal growing conditions, as the fertilizer input was not changed throughout the duration of the experiment.

Our data demonstrated that the porometer generally over-estimates *g*_s_ in growth chamber conditions (Fig. 3), and this is independently confirmed in field measurements of the same taxa and other woody angiosperms (Fig. 5). Similar to our findings, Ramírez et al. (2006) demonstrated that porometer measurements over-estimated *g*_s_ by approximately 32% compared to IRGA measurements in *Stipa tenacissima* L. The over-estimation of *g*_s_ values using the porometer is ~ 25% on average and is more pronounced in species with low *g*_s_ and under conditions that lead to decreased *g*_s_ (e.g., elevated CO_2_—Fig. 5). Yet, this difference was not statistically significant. The CO_2_ effect on the relative *g*_s_ difference is likely the result of a stomatal closing response when CO_2_ is increased, thus decreasing *g*_s_ and increasing the difference in measured *g*_s_ between the IRGA and the porometer. In addition, the relative differences in *g*_s_ are the result of the differences in how *g*_s_ is measured between the devices. The IRGA system allows more time for the leaf-chamber conditions to equilibrate before the measurement is taken (usually within 15 min.), whereas it only takes a few seconds for equilibrium to occur in the porometer chamber. Steady-state porometers are used frequently in plant studies (Jones 1999; Grant et al. 2007; Maes et al. 2016; Keel et al. 2006; Nijs et al. 1997), making the findings presented here relevant to many eco-physiologists, who rely on the accuracy of these devices. Using the proposed relationship adjustment by Murray et al. (2019) to account for this observed difference would help future comparative studies that would like to make use of measurements from both porometer and IRGA studies.

The porometer can be temporally limiting as it only provides an instantaneous measurement of *g*_s_ at a given time, whereas the IRGA can provide multiple *g*_s_ measurements over a longer time scale (up to 28 h depending on settings). When the measured species display large fluctuations in *g*_s_ across the diurnal cycle, this becomes important, as porometer protocols, which usually involve a single spot measurement per day, are less likely to capture this variability. For example, the diurnal shift in maximum *g*_s_ with elevated CO_2_ was previously suggested by Konrad et al. (2008) using an optimization model. He showed that under given environmental conditions, maximum *g*_s_ happens between 7.00 and 10.00 am for CO_2_ values < 700 ppm, but that *g*_s_ shifted to 10.00–13.00 for atmospheric CO_2_ values > 700 ppm. Similarly, we demonstrated that for *P. tremuloides* and *S. racemosa*, maximum *g*_s_ shifted into the afternoon as a result of elevated CO_2_ (Fig. 4). Short-term changes in stomatal aperture are often caused by diurnal variations of temperature, insolation, atmospheric humidity, light and wind speed (Konrad et al. 2008). ‘Long-term’ exposure in atmospheric CO_2_ on the other hand, has shown to affect stomatal anatomy and thus maximum theoretical conductance (*g*_max_) (Franks and Beerling 2009). The species-specific difference in maximum *g*_s_ to an increase in CO_2_ observed here, is likely to be a response of anatomical stomatal changes (e.g., density and size). For example, an increase in CO_2_ is often associated with a decrease in stomatal density and an increase in stomatal size (Franks and Beerling 2009). Although we did not measure anatomical traits here, Konrad et al. (2008) showed that the timing of maximum *g*_s_ is strongly dependent on the environmental conditions, stomatal traits and the rate of assimilation. It is likely that our species did adjust their *g*_max_ and thus optimized their timings of physiological responses to elevated CO_2_.

Changes of the time of the day when water is lost by plants could potentially influence the timing of precipitation in some biomes by altering plant-atmospheric dynamics, particularly in biomes where evaporation makes up a large proportion of the evapotranspiration flux (Schlesinger and Jasechko 2014). In addition, the shift in *g*_s_ with elevated CO_2_ has important consequences for the way *g*_s_ is measured in studies that are interested in the effect of elevated CO_2_ on *g*_s_. In particular it is critical to know the diurnal *g*_s_ pattern of the species to be studied, if the aim of the research is to measure maximum *g*_s_. For example, our species do not show a very strong mid-day depression, which has been observed in other taxa (Pathre et al. 1998; Franco and Lüttge 2002; Tucci et al. 2010; Kosugi and Matsuo 2006). As previously mentioned, the absolute *g*_s_ value may be overestimated, but more importantly the optimal time (i.e. *g*_s_ is at its maximum) when measurements are taken between the ambient and high CO_2_ treatments will differ as a result. For example, we found that an increase of 70 ppm CO_2_ in *S. racemosa* shifted the maximum *g*_s_ into the afternoon by approximately 1–2 h. This response can be very species-specific (Fig. 4) and is also likely to depend on the model fitted. An experiment that aims to identify how maximum *g*_s_ differs between CO_2_ treatments, using a sample protocol with fixed time-points across all treatments, is likely not to capture the actual maximum *g*_s_ in a day as a result of such a shift. It would therefore be advisable to combine porometry with 24 h response measurements from an IRGA to ensure that the measuring times between treatments are adjusted. However, it remains to be seen whether our findings are applicable across different environmental conditions and taxonomic groups.

## Conclusion

Our study demonstrated that the species in this study respond non-linearly to increases in CO_2_ concentration when exposed to decadal changes in CO_2_; small CO_2_ concentrations increases (70 ppm) often not tested by other studies. In addition, we showed that the daily maximum *g*_s_ can, in some species, shift later into the day when plants are exposed to only 70 ppm increases in CO_2_. Our findings have potential important implications to the diurnal water and carbon budget of plants, and the feedback of these across the soil–plant–atmospheric continuum; specifically under future changes in atmospheric CO_2_. Due to the importance of stomata regulating global water fluxes, CO_2_ effects that result in *g*_s_ (a) decreasing non-linearly and (b) possibly shifting diurnally, need to be considered, as shown here and elsewhere, when attempting to refine predictions of plant responses to CO_2_ across ecosystems.

### *Author contribution statement*

SB and JM conceived and designed the research. SB, CY and CE-K conducted the experiment. SB and CY analyzed the data. SB wrote the manuscript. All authors edited and approved the manuscript.

## Electronic supplementary material

Below is the link to the electronic supplementary material.

Diurnal light condition measured with the IRGA of plants grown under 350 (circle and red), 420 (triangle and green) and 560 ppm (square and blue) CO_2_. No IRGA data for the 490 ppm treatment was collected due to access restriction to equipment. Each value is the mean of approximately ten measurements per treatment (*n* = 10). Vertical bars represent the 95% confidence interval (TIFF 1187 kb)
