# Supplementary figures and images for: Plant responses to decadal scale increments in atmospheric CO2 concentration: comparing two stomatal conductance sampling methods
Source: Planta. 2020 Jan 16;251(2):52. doi: 10.1007/s00425-020-03343-z (PMC6965045; doi:10.1007/s00425-020-03343-z)

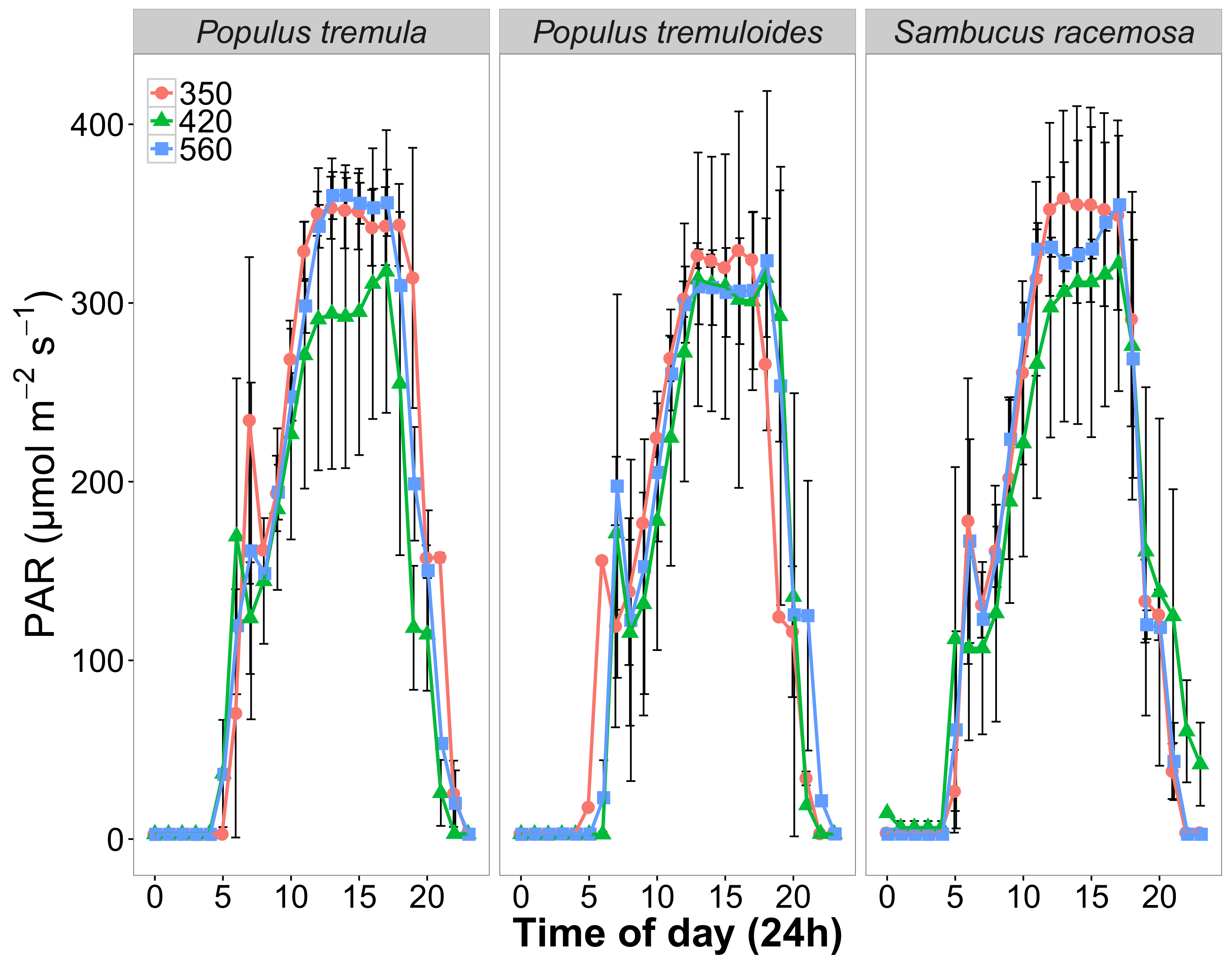

Supplement: Supplementary file 1 — Diurnal light condition measured with the IRGA of plants grown under 350 (circle and red), 420 (triangle and green) and 560 ppm (square and blue) CO2. No IRGA data for the 490 ppm treatment was collected due to access restriction to equipment. Each value is the mean of approximately ten measurements per treatment (n = 10). Vertical bars represent the 95% confidence interval (TIFF 1187 kb) [file 425_2020_3343_MOESM1_ESM.tiff]
